# Mutations in both SAMD9 and SLC19A2 genes caused complex phenotypes characterized by recurrent infection, dysphagia and profound deafness – a case report for dual diagnosis

**DOI:** 10.1186/s12887-019-1733-y

**Published:** 2019-10-21

**Authors:** Yan Zhang, Yi Zhang, Victor Wei Zhang, Chunyi Zhang, Hongke Ding, Aihua Yin

**Affiliations:** 1 Center for Medical Genetics, Guangdong Women and Children Hospital, 521 Xingnandadao, Guangzhou, 511442 China; 2 Euler Genomics Co. Ltd., Beijing, China; 3 AmCare Genomics Laboratory, Guangzhou, China; 4 Department of Molecular and Human Genetics, Baylor College of Medicine, Houston, TX USA; 5 Neonatology Department, Guangdong Women and Children Hospital, Guangzhou, China

**Keywords:** High-throughput nucleotide sequencing, Tumoral calcinosis, Normophosphatemic, Familial, MIRAGE syndrom, Thiamine responsive megaloblastic anemia syndrome

## Abstract

**Background:**

Phenotypic difference is general in Mendelian disease. Due to the extremely low incidence for a single disease, phenotype spectrum needs to be expanded. Meanwhile, earlier knowledge says patients who suffered from two kinds of different Mendelian disease are very rare.

**Case presentation:**

We describe a case of neonatal male with genital anomalies, growth delay, skin hyperpigmentation, chronic lung disease with recurrent infection, anemia, and severe deafness. Without any clear etiology after routine workflow, whole exome sequencing was carried on. A pathogenic de novo *SAMD9* mutation and compound heterozygous likely-pathogenic variants in *SLC19A2* were identified. Some symptoms were improved after the patient was treated with vitamin B1. Unfortunately, the boy died from sepsis and multiple organ failure before 1 year old.

**Conclusion:**

Combining the phenotype and clinical progress of treatment, we report that it is the first case of a patient with both MIRAGE syndrome and TRMA syndrome.

## Background

Many neonatal patients with suspected genetic causes are not given a clear diagnosis after the workflow of routine clinical practice such as the recognition of phenotypes, imaging studies or biochemical tests even with biopsy investigations. Genetic tests such as karyotyping, chromosome microarray or the targeted gene panel testing are often carried out in a stepwise approach to search single genetics etiological cause, while many patients remain undiagnosed [1]. Undiagnosed patients may miss the potential treatments, as well as, unable to evaluate the recurrence risk in subsequent pregnancies. A costly and lengthy process for resolving such rare and complicate cases, is commonly called “diagnostic odyssey” [2]. It means huge burdens for families and our medical system.

The next-generation sequencing technology has proven to be an effective way in establishing causes for many rare genetic diseases [3, 4]. Mendelian diseases are estimated to occur at a rate of 40 to 82 per 1000 live births [5]. What is more important is that recent studies show that up-to 6% patients have more than one kinds of genetic disorder [5, 6]. With the medical exome test, we can help increase the diagnosis rate to 50% (data not show) in patients with suspicious Mendelian disease. Here we report a boy with pathogenic de novo mutation in *SAMD9* and likely-pathogenic, compound heterozygote mutations in *SLC19A2*. Since newborn, the boy suffered from recurrent infection, anemia and severe deafness. So far, it is the first case of a patient with both MIRAGE syndrome and TRMA syndrome.

## Case presentation

### Patient

The Chinese Han male patient was born at 31 weeks gestational age by a spontaneous vaginal delivery, with 1000 g ± 0.2 birth weight. At birth, Apgar scores were 7, 9 and 10 at 1 min, 5 min and 10 min respectively. The patient’s mother was 33 years old with polycystic ovarian syndrome. No information regarding the father had been available. Although the entire pregnancy was uneventful without complications, the fetus was found to be IUGR. Right after the birth, the baby presented some dysmorphic features, such as dysphagia, recurrent infection, as well as hyperpigmentation, excessive body hair and thick eyebrows. Some of the facial appearances faded away gradually with growth. Hypoplastic genitalia was noticed to be female like but chromosome test turned out 46, XY. The penis is visible, while testis and scrotum are not. Ultrasound was carried on at 10 months old, which showed the left testicle is in the inguinal canal while the right is in right lower pelvic cavity. The bilateral epididymis was undetected. No solid mass was found in bilateral scrotum.

Recurrent fever occurred since 3 months old, but sometimes, the results of routine blood testing for inflammation, infections and autoimmune diseases were unremarkable and no obvious clinical features of sepsis were observed. Neutropenia and clinical features of sepsis developed during the 6 months. Due to respiratory distress and chronic lung infection, mechanical ventilation was implemented for about 2 months. *Escherichia coli*, *Klebsiella pneumoniae* and *Stenotrophomonas maltophilia* were found separately via sputum culture. Anomalous pulmonary venous drainage was detected at 6 months and surgery was performed, after which nearly normal cardiac function was restored.

**Fig. 1:**
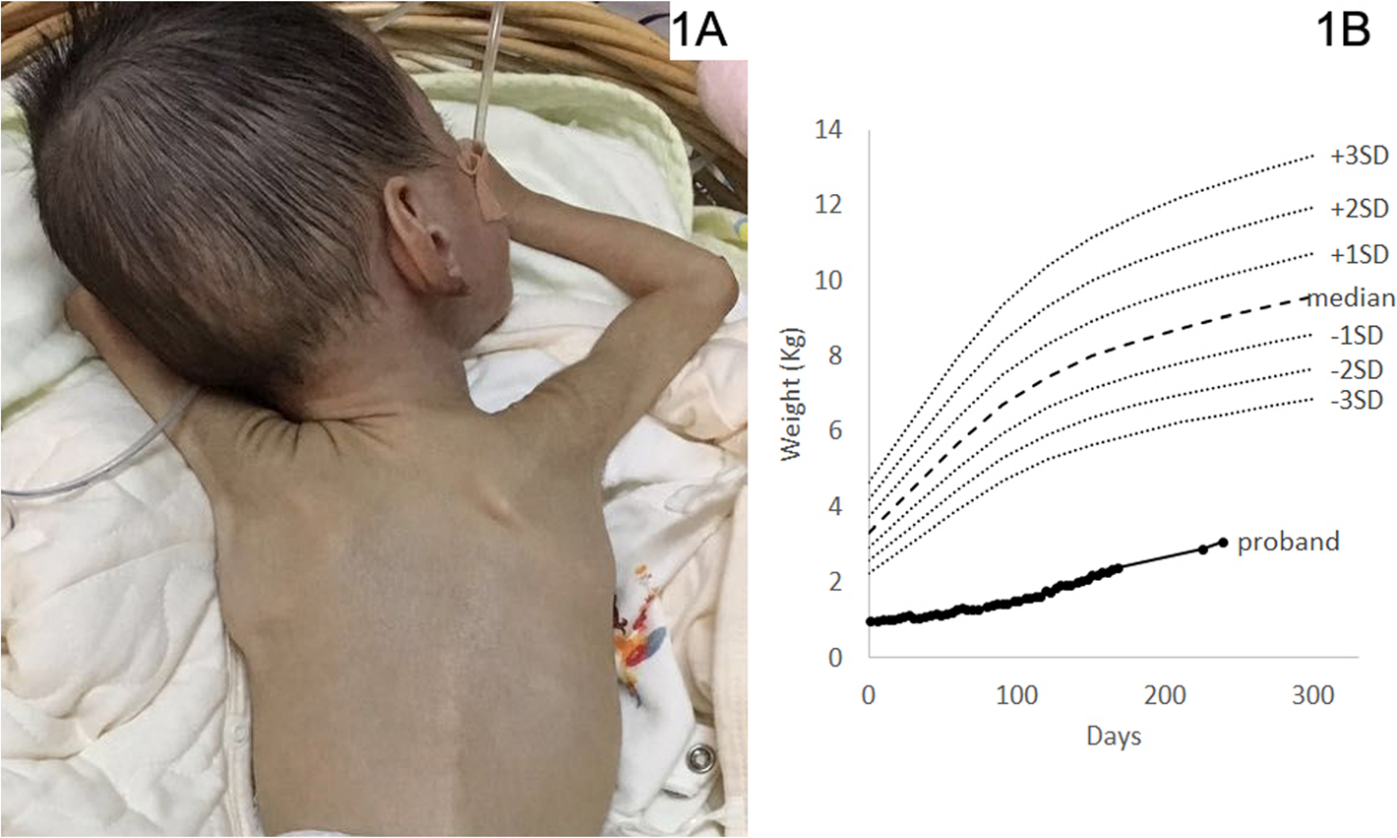
Photograph and growth curve of the proband. **a** Photo of the proband’s back at 2 months, he is extremely thin and skeletonizing. **b** The proband with low birth weight grew slowly and lagged obviously behind the referent range [7]

Enteral nutrition was initiated at the third day after birth since the patient has difficultly to suck and swallow. He still suffered from severe growth retardation (less than 3SD) even enteral feeds was given by tube > 150 mL/kg/d and daily calories intake > 130 kcal/kg/d. He had severe malnutrition (weighed 3000 g) and presented chronic diarrhea when enteral feeds were increased. He got severely developmental delay and did not meet the growth milestones at 10 months old (unable to lift his head well or sit alone) (Fig. 1a and b [7]).

Thrombocytopenia and anemia developed since 2 months old and blood transfusion was initiated. Because of matched manifestations such as short status, hyperglycemia, and anemia, vitamin B1 was tried to use to cure the infant. Whilst megaloblastic anemia was partially controlled, granulocytopenia was not.

Since 3 months old, there were recurrent onsets of seizures, no abnormal findings in repeated cerebral spinal fluid for biochemical and pathogen testing. Magnetic resonance imaging showed poor myelination while electroencephalogram did not indicate any remarkable sign. Auditory brainstem response test showed moderate-severe deafness. Blood gas analysis showed no acidosis. Glucose and electrolyte levels were within normal range.

There was no agenesis phenotype in adrenal glands detected by ultrasonography. Plasma cortisol was 18.1 μg/dL (referent range 5-25 μg/dL) while plasma ACTH was 29.7 pmol/L (referent range 0-10.21 pmol/L).

Finally, the patient died from sepsis at 12 months. Recurrent fever, recurrent seizure, malnutrition, developmental delay and chronic diarrhea persisted during the last several months.

### Genetic testing

Peripheral blood samples were drawn from the infant and his parents after the informed consent. Genome DNA was prepared with magic beads (Zeesan Biotech, Xiamen, China). Sequencing libraries were prepared with standard TruSeq protocol and followed by liquid phase exome capture and were sequenced with Illumina HiSeq 2000. We analyzed sequencing data with standard practice. Briefly, raw sequencing reads were aligned to GRCh37+ decoy genome with BWA and processed with sambamba view. Variant calling was done with Sentieon and further filtered by Freebayes. Filtered variants were annotated with SnpEff and filtered against population frequency (gnomAD and Euler Genomics in-house Han Chinese population database). Mendelian transmission types of mutations were determined by standard practice. Sanger sequencing was used to verify the de novo *SAMD9* mutation by mutant sequence-specific primers (PF: 5’GACTTGACCCAGTGTATCTG3’ and PR: 5’GTCTATC TTCTGCAGTACTC3’).

**Fig. 2:**
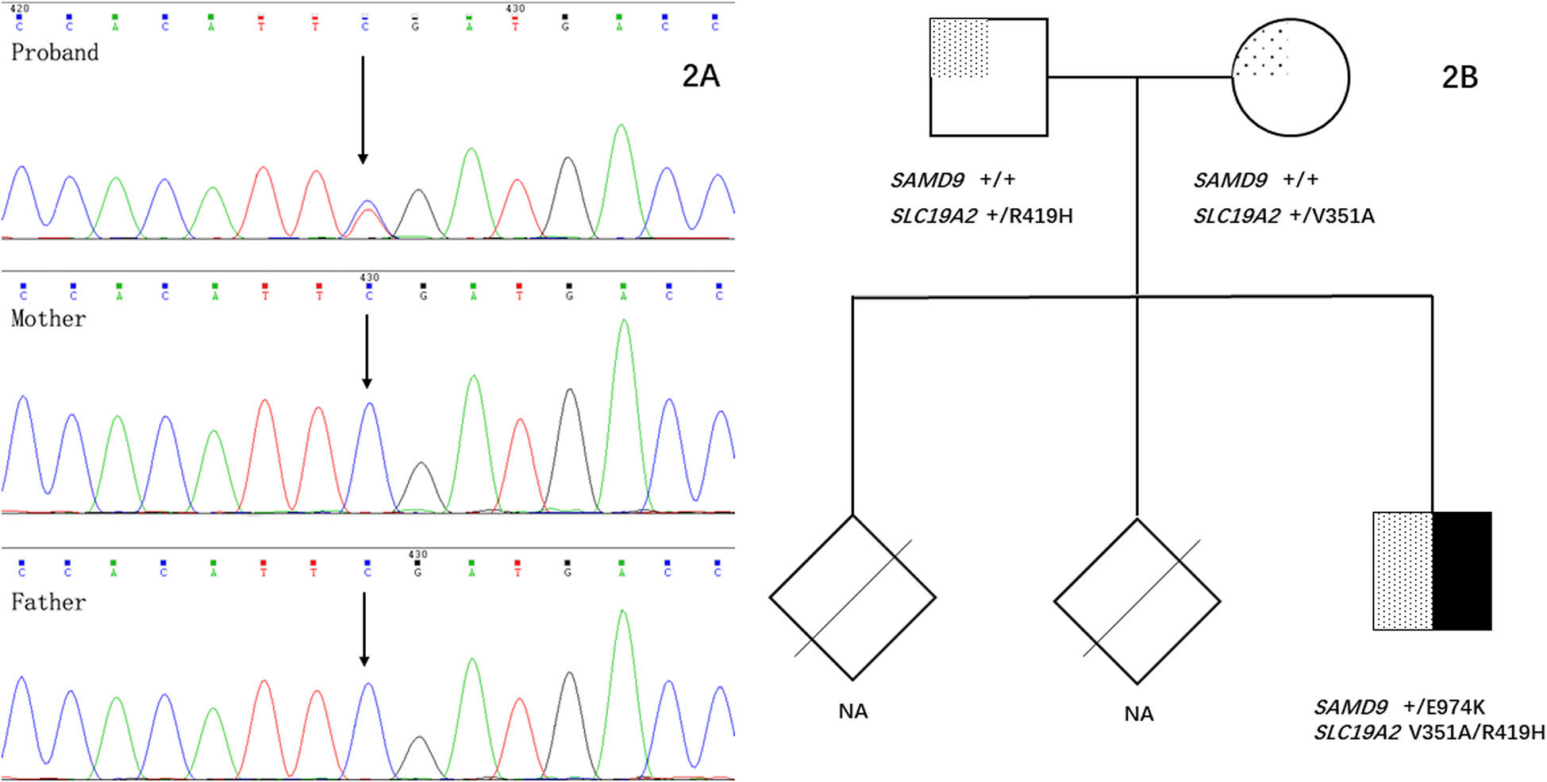
Pedigree and verification of the de novo variation in *SAMD 9* in the family. **a** The proband has a heterozygote variant but the parents not. **b** Pedigree of the patient is shown with *SAMD9 / SLC19A2* genotype information. Black square indicates the patient affected by MIRAGE syndrome caused by a de novo variant in *SAMD9*. Rhombus indicate stillbirth. NA denotes genotype not available. Square filled with dots indicates the proband suffered from TRAM syndrome. The compound heterozygote found in the patient was carried by father and mother respectively

Filtering against 0.1% population frequency and functional annotation revealed a pair of *SLC19A2* (NM_006996) compound heterozygous mutations in the boy (c.1256G > A (p.R419H), rs530420883, paternal origin and c.1052 T > C (p.V351A), rs748588472, maternal origin), as well as a de novo heterozygous missence mutation c.2920 G > A (p. E974K) in *SAMD9* gene (NM_017654) (Fig. 2a, b).

All three variants are not recorded in open databases (HGMD, ClinVar and UniProt). They affect highly conserved amino acid residues (UCSC) and are predicted to be disease causing (mutationtaster), damaging (SIFT) or probably damaging (Polyphen 2). Neither c.1256G > A (p.R419H) nor c.1052 T > C (p.V351A) in *SLC19A2* was reported earlier, allele frequencies were around 0.00002 and 0.00009 respectively (gnomAD). Most heterozygotes of c.1052 T > C (p.V351A) were from east Asia showed in gnomAD. The variant was found in both healthy individuals and tumor patients in China from DiseaseDX database (https://diseasedx.virgilbio.com/). The compound heterozygotes in SLC19A2 gene were first reported here in TRMA associated disease.

Additionally, the *SAMD9* mutation c.2920 G > A (p.E974K) has been reported as pathogenic in a patient with MIRAGE syndrome previously [8].

## Discussion and conclusions

With whole exome sequencing, we found one heterozygous variant in *SAMD9* and two compound heterozygous mutants in *SLC19A2*.

*SAMD9* was known to be associated with MIRAGE syndrome, which is a newly reported disease (OMIM ID: 617053) [8, 9]. The core symptoms were adrenal hypoplasia, developmental delay, genital abnormality, anemia, recurrent infection, chronic diarrhea. Reports show the full clinical spectrum of *SAMD9* defects still need to be determined [10, 11]. Adrenal hypoplasia has been the most consistent manifestation of the MIRAGE syndrome. The synopsis was seen in 19 out of 21 evaluated patients with MIRAGE syndrome [8, 11, 12]. This fact highlights the significance of our patient’s lacking adrenal features. One patient in Federica Buonocore’s report [12] had no adrenal insufficiency who has a frame shift mutation in *SAMD9.* Also, one patient in Satoshi Narumi’s research had normal-sized adrenal gland despite a high plasma corticotropin level [8]. The normal ultrasound result for adrenal and higher plasma cortisol reappeared in our patient. SAMD9 is widely expressed and is regulated by TNF-alpha. The protein may play a role in inflammatory response and the control of extra-osseous calcification. Research shows *SAMD9* associated abnormality was caused by gain-of-function mutation. In the affected fibroblast cells endosome organization changed [8]. The reduced cell proliferation could be rescued by another loss-of-function mutant in *SAMD9* [12]. Maybe there are some other unknown regulated variant in both our patient and the Satoshi Narumi’s patient. Our experience verified the variable expressivity of the adrenal phenotype in MIRAGE syndrome.

c.2920 G > A (p.E974) in *SAMD9* has been reported to be pathogenic in a patient with MIRAGE syndrome previously [8]. MIRAGE syndrome matched multiple clinical manifestations in the patient include myelodysplasia, infection, the restriction of growth, genital phenotypes, enteropathy, poor sucking capability, dysphagia, and choking. On the basis of the same mutation and similar synopsis with earlier report [8], we can make the diagnosis of MIRAGE syndrome for the infant. TRMA syndrome is characterized with sensorineural deafness, developmental delay, seizures, megaloblastic anemia, sideroblastic anemia (OMIM ID: 249270)[ 13, 14]. The syndrome is caused by *SLC19A2* which encodes Thiamine transporter 1(THTR-1), which is co-expressed with THTR-2 in most tissues except bone marrow, pancreas islet and cochlear cells. THTR-2 acts as a compensate of THTR-1 when transporting thiamine. Pathogenic compound heterozygotes or homozygotes could damage the function of THTR-1, while clinical synopsis mainly occurred in hearing, blood glucose and hematopoiesis [15].

c.1256G > A (p.R419H) and c.1052 T > C (p.V351A) in *SLC19A2* are not reported earlier. These two mutations were recorded with allele frequency were 0.00002, 0.00009 in gnomAD and 0, 0.0005 respectively in Chinese (http://diseasedx.Virgi lbio.com/variation/list?id=01f1351e5929ca5c84a545ac59f4027d&table=create1000GenomeDisease&variationType=part1proteins&version=38). p.V351A locates in the transmembrane region of THTR-1 (338th to 354th amino acid). The mutant residue is smaller than the wild-type residue. This size difference can affect the contacts with the lipid-membrane. Thus, change the transmembrane domain. p.R419H is in the cytoplasmic topological domain (410th to 419th amino acid). This variant changed the size of amino acid and the charge and might lead to changing interactions with other molecules or residues [16].

The compound heterozygotes and the associated phenotypes made the diagnosis of TRMA well documented. After supplementation with thiamine for one-month, clinical manifestations of anemia and granulopenia got better. In combination with the gene mutation, we speculated that anemia in the proband might be caused by the *SLC19A2* mutations resulting in THTR-1 dysfunction.

There is no report about the relation between *SAMD9* and *SLC19A2* either their expressed products*.* The associated clinical synopsis such as myelodysplasia, seizure, anemia, granulopenia and infection may be caused by separating mechanisms.

As a conclusion, we found the patient suffered from both TRMA and MIRAGE syndromes. Coincidences of two Mendelian diseases in one patient are still very rare, but these cases can be found by next-generation sequencing. The medical exome test is a useful tool when face to undiagnosed patients and could be used as an effected alterative in genetics lab.

## Data Availability

The datasets generated and/or analyzed during the current study are available in the [28875-28877] repository, [https://pan.baidu.com/s/1P6c8i9zv5L9JA8tjCAfUfQ/j0k1].

## Abbreviations

HGMD: Human Gene Mutation Database
IUGR: Intrauterine growth retardation
MIRAGE: Myelodysplasia, infection, restriction of growth, adrenal hypoplasia, genital phenotypes, and enteropathy
OMIM: Online Mendelian Inheritance in Man
TRMA: Thiamine-responsive megaloblastic anemia
UCSC: University of California, Santa Cruz Genome Browser Home
